# Differences in non-positive intention to accept the COVID-19 booster vaccine between three countries in the cross-border region Meuse-Rhine Euroregion: The Netherlands, Belgium, and Germany

**DOI:** 10.1016/j.jvacx.2023.100306

**Published:** 2023-04-20

**Authors:** Céline J.A. van Bilsen, Christina Stabourlos, Chrissy P.B. Moonen, Stephanie Brinkhues, Stefaan Demarest, Daniëlle A.T. Hanssen, Inge H.M. van Loo, Paul H.M. Savelkoul, Dirk Philippsen, Brigitte A.M. van der Zanden, Nicole H.T.M. Dukers-Muijrers, Christian J.P.A. Hoebe

**Affiliations:** a Department of Social Medicine, Maastricht University, Care and Public Health Research Institute (CAPHRI), Maastricht, the Netherlands; b Department of Sexual Health, Infectious Diseases, and Environmental Health, Public Health Service South Limburg, Heerlen, the Netherlands; c Department of Epidemiology and Public Health, Sciensano, Brussels, Belgium; d Department of Knowledge & Innovation, Public Health Service South Limburg, Heerlen, the Netherlands; e Department of Medical Microbiology, Infectious Diseases & Infection Prevention, Maastricht University Medical Center (MUMC+), Maastricht, the Netherlands; f Care and Primary Health Research Institute (CAPHRI), Maastricht University, Maastricht, the Netherlands; g Gesundheitsberichterstattung, Gesundheitsamt Düren, Düren, Germany; h Foundation euPrevent, Heerlen, the Netherlands; i Department of Health Promotion, Care and Public Health Research Institute (CAPHRI), Maastricht University, Maastricht, the Netherlands

**Keywords:** COVID-19, COVID-19 vaccines, Pandemics, Surveys and questionnaires, Logistic models, Intention

## Abstract

COVID-19 booster vaccination has shown to add to the protection against infection with SARS-CoV2 and subsequent severe disease. This longitudinal cross-border study aimed to identify factors associated with COVID-19 booster vaccine intentions in an initially vaccinated adult population living in the Meuse-Rhine Euroregion (EMR; including the Netherlands, Belgium, and Germany) and differences between countries.

Data collection took place in autumn of 2021 and consisted of online questionnaires sent to a random sample of the population based on governmental registries. Data from 3,319 fully and partially vaccinated adults were used to examine determinants of non-positive intention for a booster vaccination (i.e., uncertain or do not want), using multivariable logistic regression analyses weighted by age group, sex, and country.

Compared to German residents, Dutch residents (OR = 2.4) and Belgian residents (OR = 1.4) were more likely to be uncertain or not want to receive a booster vaccine in September-October 2021. Factors independently associated with non-positive intention were female sex (OR = 1.6), absence of comorbidities (OR = 1.3), time since last vaccination less than 3 months ago for those fully vaccinated (OR = 1.6), being partially vaccinated (OR = 3.6), a negative experience with communication of COVID-19 measures (OR = 2.2), and regarding measures as ineffective (OR = 1.1).

Results indicate that booster vaccine intentions differ between countries in the cross border Meuse-Rhine Euroregion. Non-positive intention for the booster vaccine is prevalent in all three countries of the EMR, but to a different extent, as shown in this study. Cross-border collaboration and sharing information and knowledge about vaccination strategies could play a role in limiting the impact of COVID-19.

## Highlights

• This cross-border study examined determinants of non-positive intention for COVID-19 booster vaccination in the EMR.
• In autumn 2021, non-positive intention ranged from 15% (Germany) to 20% (Belgium) and 32% (Netherlands).
• Differences between countries highlight the need for cross-border collaboration to limit the impact of COVID-19..

## Introduction

In 2021, vaccination campaigns worldwide were a breakthrough in the battle against coronavirus disease 2019 (COVID-19) [1]. However, with the emergence of new variants it became apparent that later in the same year, COVID-19 booster vaccination would become necessary. Since antibody response declines over time and new variants can be more transmissible and evade immune responses, boosters provide additional protection [2], [3]. COVID-19 vaccination coverage was already challenging, but to keep the situation manageable and with new variants emerging, booster hesitance is the next challenge.

Bivalent vaccines by BioNTech/Pfizer and Moderna are to be used in the autumn of 2022 and will be offered to adults to reduce the hospitalizations and deaths. These vaccines contain the mRNA components of both the Wuhan and the omicron variant. Apart from mRNA vaccines, Janssen and Novavax offer booster vaccines. All 4 types of booster vaccines are approved by the European Medicines Agency (EMA) [4], [5]. While moving to a context where booster vaccination is expected to remain important, it is crucial to understand attitudes towards booster vaccination.

Different countries have approached vaccination differently, potentially resulting in different uptakes [6]. For example, a cross-sectional study conducted in Croatia in December 2021 showed a percentage of 79% for positive intention among vaccinated participants [7]. Higher percentages for intention were determined by Rzymski et al. (Poland, 82.3%) and Yadete et al. (United States of America, 87.1%), who collected data from vaccinated participants in July 2021 and September 2021, respectively [8], [9]. An observational study of 22,139 fully vaccinated adults in the UK also examined predictors of uncertainty and unwillingness to receive a COVID-19 booster vaccine in November-December 2021 and showed a 4% rate of unwillingness and a 4% rate for uncertainty [10]. Distinct factors have been associated with booster vaccine intentions, such as age, sex, and risk perception. In several studies, young adults and women were more likely to have a non-positive intention for booster vaccination [8], [11]. Young adults may be more hesitant about accepting a booster vaccine because they generally have lower risk perception of being severely affected by the disease [12]. Likewise, people without comorbidities generally are less favorable towards booster vaccination [10], [13], which might suggest that they have a lower risk perception and are therefore less likely to take the booster vaccine.

It is not known what factors are associated with booster vaccine intention in a border region such as the Euroregion Meuse-Rhine (EMR), which is the border region of the Netherlands, Belgium, and Germany. The population of the different regions, in total 4 million, is relatively homogeneous in terms of age distribution, social-economic situation, lifestyle, and health profile. Because of the open-border nature of the region, normally most inhabitants do not feel borders exist in daily life. However, during the COVID-19 pandemic the disparity in approaches to tackle the situation between countries became apparent [14]. In September 2022, the percentage of the adult population that had received at least one COVID-19 vaccine was 91% in Nord Rhine-Westphalia, to which the German part of the EMR belongs, between 86% (Ostbelgien and Wallonia, to which Liège belongs) and 95% (Flanders, to which Belgian Limburg belongs) in the Belgian part of the EMR, and 81% in South Limburg, the Dutch part of the EMR [15], [16], [17].

While booster vaccination is expected to remain important, pandemic fatigue and vaccine hesitancy form a threat for global health [18], [19]. Therefore, it is relevant to understand the handling and effects of booster campaigns in different countries in order to share knowledge and inform policy optimization. Here, we aimed to identify factors associated with COVID-19 booster vaccine intentions in the fully and partially vaccinated adult population living in the EMR and to identify differences between countries. Given that the uptake of the initial COVID-19 vaccine differed between countries, we hypothesized that intentions for booster vaccination would also differ between the three EMR-countries. Additionally, we expected previously established factors such as age, sex, and presence of comorbidities to be associated with booster vaccine intention.

## Methods

### Context of the study

Data on booster intention were collected in September-October 2021, when the Delta variant was dominant and at its peak [20].

#### The Netherlands

In the Netherlands, several measures were in place in this period: the advice to work from home, closing of night clubs, and the obligation to make reservations and register for visiting restaurants, hotels, and cafes [21]. Booster vaccination in the Netherlands started in the end of November 2021 for vulnerable groups, elderly, and healthcare workers. In January 2022, booster vaccination also became available for other adults [22], [23].

#### Germany

The German regions of the EMR all belong to the state of Nordrhein-Westfalen. In September-November 2021, masks were obligated and a 3G (negatively tested, vaccinated, or recovered from a recent infection) policy was implemented for visiting events, restaurants, and facilities when the national 7-day incidence would exceed 35 [24]. In Germany, booster vaccination for elderly people and healthcare workers was available from October 2021, and was recommended for all adults from the end of November 2021 [25], [26].

#### Belgium

In Belgium, most measures that were enforced during summer, were relaxed in September 2021 [27]. Masks were still obligated in public transport and certain places of activity. However, at the end of October 2021, changes regarding masks were reversed again, working from home was strongly encouraged, and the “Covid safe ticket” (QR code) for events was introduced [28]. In September 2021, most elderly and vulnerable groups could receive the booster vaccine. From December 2021 onwards, the capacity further increased so that the majority of adults could receive the booster [29], [30].

### Study design and participants

This study is part of a larger longitudinal study in inhabitants of the EMR: ‘Impact of COVID-19 on the EMR’. An interregional partnership was established to study the impact of the pandemic in a cross-border setting by using comparative data collection. Data were collected in April-June 2021 (round 1) and September-November (round 2) 2021, and consisted of two self-finger prick antibody tests and two online questionnaires, available in Dutch, German, and French. The questionnaire included information on general health, COVID-19 infection and symptoms, booster vaccine intention, cross-border mobility changes, infection prevention attitudes and behavior, and loneliness. An online tool was used to manage fieldwork procedures and monitor participation and antibody test results, with a multilingual helpdesk set up to support participants. A complete overview of the sampling, design and participation is described elsewhere [31].

**Fig. 1:**
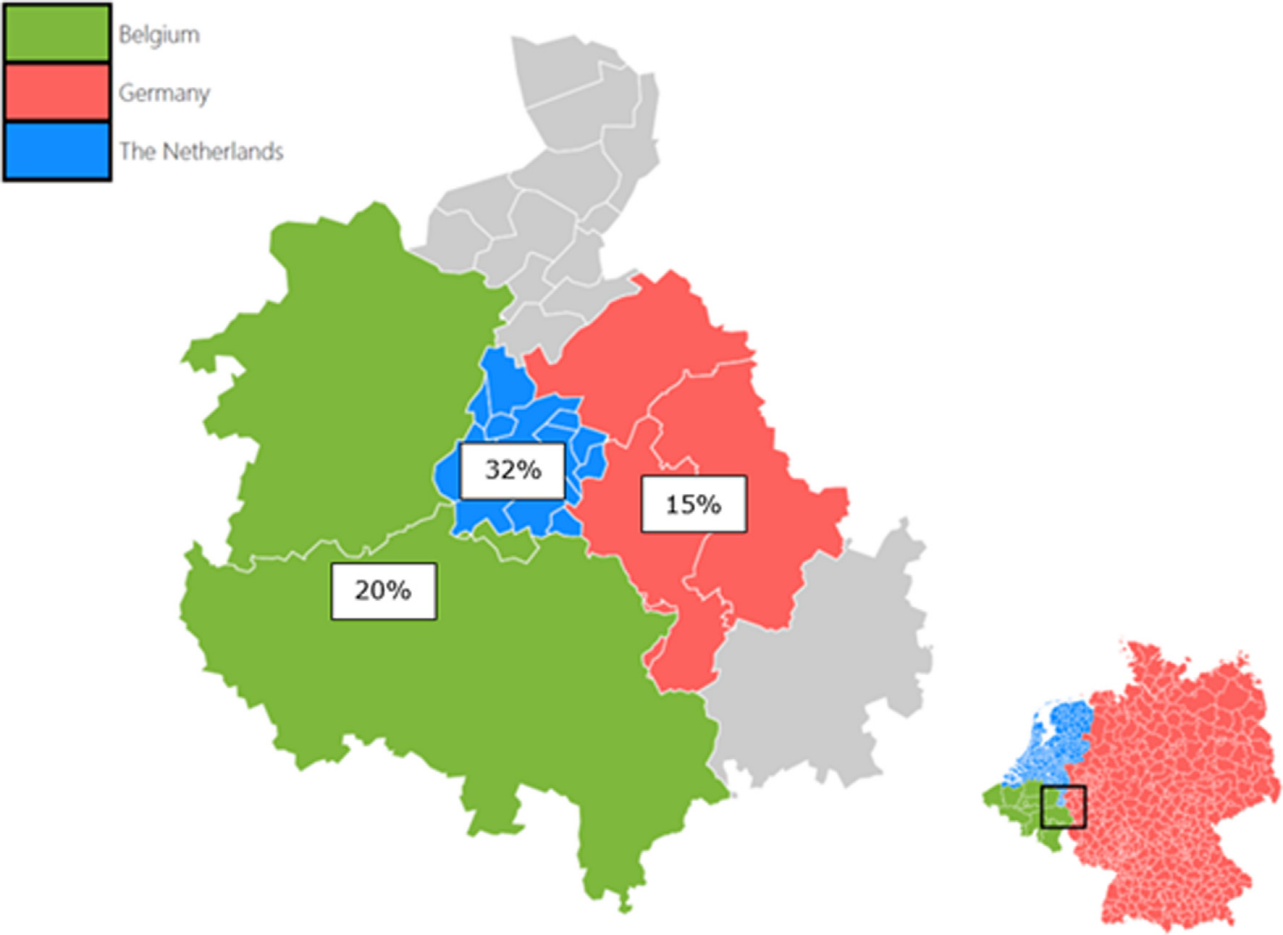
Non-positive intentions for booster vaccination in the Meuse-Rhine Euroregion in autumn 2021 (weighted percentages).

The Meuse-Rhine Euroregion covers the border area between Belgium, the Netherlands and Germany. The subregions included in this study are the Belgian provinces Limburg and Liège (including the German-speaking community Ostbelgien), South Limburg (the Netherlands), and the German City Region Aachen and Districts of Düren and Heinsberg (Fig. 1). Citizens of 18 years and older and residing in private households in the EMR were selected from the national registers in each country. A random sample was selected from the latest version of the National Register (NR) in Belgium, the Basic Registration of Persons (BRP) in The Netherlands, and the Registration Offices (Einwohnermeldeämter) in Germany. The sample size was set at 3,500 in each country, which was distributed among the sub-regions based on the size of the adult population. The included adult population was classified according to age group and sex. After ordering, a stepwise selection was applied, where the step size in each sub region was based on the total eligible population of that sub region divided by its sample size, starting from a random number. In Germany, the citizens from District Heinsberg and City Region Aachen were only classified by sex and a randomized sample was taken. Invitations were sent in the official language of the sub-region, along with an information brochure, personal ID-code, link to the online questionnaire, and informed consent form. Detailed information about the sampling methods applied in the three different countries, stepwise selection, and opt-out procedures can be accessed elsewhere [31].

Round 1 participation rates differed from 15.3% in the Belgian subregions (8,911 invitees and 1,366participants) to 23.7% (6,748 invitees and 1,598participants) and 27% (11,266 invitees and 3,042 participants) in the German and Dutch subregions, respectively. In round 2, participants of round 1 were invited (N = 6,006). Full participation in both rounds was 3,344.

### Current analyses

In the current study, complete case analysis was used. Participants who filled out both questionnaires, were fully or partially vaccinated at the time of round 2 data collection, and had no missing data were included. Fully vaccinated was defined as having received 2 vaccines, 1 vaccine in the case of Janssen vaccine, or a previous SARS-CoV2 infection plus 1 vaccine. Data on COVID-19 booster vaccination intention were collected between September 21 and November 22, 2021. Results from this group of participants are described in this paper.

### Variables

#### Outcome

Intention to receive a COVID-19 booster vaccination was measured via the question: ‘Do you intend to take the booster vaccine, once it is available?’. Response options were ‘Yes’, ‘No’, or ‘Not sure yet’. The primary outcome is booster vaccine intention, regrouped into: ‘positive intention [reference group]’ versus ‘negative/neutral intention [no and not sure yet]’.

#### Personal factors

Various socio-demographic factors were included: country of residence, (including Germany, Belgium, and the Netherlands), sex (male or female), age group (18–59 or ≥ 60 years), and level of education (theoretical or practical). Employment status was categorized into 5 categories: retired, working in healthcare, working in education/childcare/youth assistance, working in another sector, not working or student. To measure interpersonal trust, one item of the de Jong-Gierveld scale for loneliness was used: “There are many people that I can count on completely”. Response options were (totally) agree, neutral or (totally) disagree. In the first round of the study, presence of comorbidities (yes or no) was assessed.

#### COVID-19 related factors

Participants reported whether they had a previous COVID-19 infection (yes or no). Time to last COVID-19 vaccination at the time of questionnaire completion was included as a factor and was divided in 3 groups: fully vaccinated with the last vaccination received ≤ 3 months ago, fully vaccinated with the last vaccination received >3 months ago, and partially vaccinated. Apart from that, the questionnaire included an item about the experience of the communication of measures: “Please indicate the extent to which you agree with the following statement: I have found the communication of infection prevention measures to be good. Think about communication from the government through the news, television, newspaper and radio”. Response options were (totally) agree, neutral, (totally) disagree. Finally, participants were asked in round 2 whether they find COVID-19 infection prevention measures useful or effective, including social distancing, washing hands with water and soap, sneezing and coughing in the elbow, wearing a mask, restricting group size at home, minimalizing travel, working from home, requiring a QR code for attendance of activities, and testing and staying home in case of symptoms. Response options ranged from 1 (very effective) to 7 (not effective at all). A score was calculated by adding all responses together, where a high score indicates regarding measures as lowly effective.

### Weighted results

To increase representativeness of the study for the general population, data were weighted to the proportions of country (the Netherlands, Belgium, and Germany), age group (18–29, 30–39, 40–49, 50–59, 60–69, 70–79 or ≥80 years), and sex (male or female) obtained from national registers. Weigh factors were calculated taking into account non-response and selection probability for each of the age- and sex strata per country.

Weigh factors were calculated for each of the 3 EMR-regions separately (the Netherlands, Belgium, and Germany) per age and sex stratum (male/female and 18–29, 30–39, 40–49, 50–59, 60–69, 70–79, 80–89, and 90+ years). Sex and age distributions of the total populations were obtained from governmental registries. Composite weigh factors were used in the statistical analyses to obtain weighted results. These weigh factors were calculated by multiplying the non-response weights and the post-stratification weights. All weigh factors had a mean value of 1.

Non-response weights were obtained by dividing the response numbers by the number of citizens invited. The reciprocal of this was multiplied by the total response in the age and sex stratum. This number was then divided by the total number of invitees of the age and sex stratum. Post-stratification weights were calculated by taking the reciprocal of the selection probability (invitees divided by number of citizens in population) multiplied by the total number of invitees in the age and sex stratum. This value was then divided by the total number of citizens in the population of the age and sex stratum.

### Statistical methods

After calculating weighted proportions with 95% confidence intervals (95% CI), weighted univariate and multivariate logistic regression analyses were performed to assess factors associated with intention to receive a COVID-19 booster vaccination. The multivariable model was adjusted for the confounders sex, age group, and level of education. Other variables with a two-sided p-value of <0.10 in univariate analyses were included in the multivariable model. With these variables, a backwards selection procedure was applied to create the final multivariable model (while always keeping the confounders in the model). A two-sided p-value of <0.05 was considered statistically significant. Data were analyzed with IBM Statistical Package for the Social Sciences (SPSS) version 27.

### Missing data

Questions in the questionnaire were not compulsory, leading to the possibility of missing data. Participants with missing data for at least one of the predictor variables were excluded from the analysis.

### Multicollinearity and interaction

There was no multicollinearity since all Variance Inflation Factor (VIF) values were < 3. We tested for statistical interactions (effect modification) of sex and age group with country. Sex showed a statistically significant interaction (p < 0.10) with country. However, stratified analysis by country showed that odds for non-positive booster intention by sex slightly differed per country and that in each country the intention to take the booster vaccine was higher among men. Therefore, results presented in this article were not stratified. There was no statistically significant interaction between age group and country.

## Results

### Participants

**Fig. 2:**
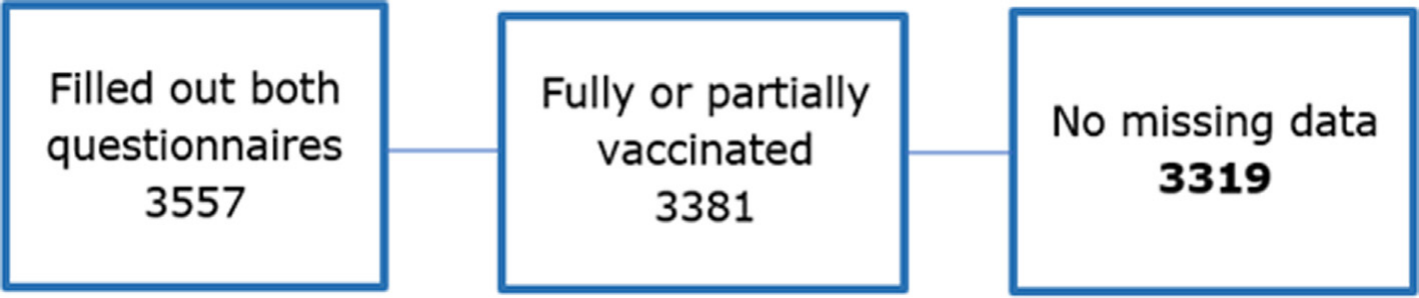
Participation and formation of the study population.

Missing data resulted in exclusion of 62 participants, totaling in a final N of 3,319 (Fig. 2). Neutral intention of taking the booster vaccine was expressed by 601 participants, whereas negative intention was expressed by 211 participants.

### Booster intention proportions

Weighted proportions for booster intention are presented in Table 1. Non-positive intention differed per country and ranged from 14.6% in the German subregion to 19.8% in the Belgian subregion and 32.1% in the Dutch subregion (Fig. 1). In Belgium, notable differences between the different provinces were observed, where non-positive intention ranged from 14% in Limburg to approximately 30% in Liège and Ostbelgien.

**Table 1:** Proportions of non-positive intention by personal factors and COVID-related variables.

		**Non-positive intention**		
		N (total)	Weighted %	95% CI
**Country**	Germany	150 (985)	14.6	12.5–16.9
	Netherlands	526 (1655)	32.1	29.9–34.4
	Belgium	136 (679)	19.8	16.9–22.9
**Age group**	18–59	567 (1872)	25.9	23.5–28.5
	≥60	245 (1447)	13.1	11.0–15.6
**Sex**	Male	239 (1376)	16.2	13.7–18.9
	Female	573 (1943)	23.3	21.0–25.7
**Level of education**^a^	Theoretical	360 (1565)	21.4	18.8–24.2
	Practical	452 (1754)	19.3	17.1–21.7
**Comorbidities**	Yes	434 (2017)	17.3	15.3–19.4
	No	378 (1302)	25.2	22.3–28.4
**Work situation**	Retired	130 (907)	11.4	9.0–14.3
	Working in healthcare	129 (370)	30.9	25.0–37.5
	Working in education, childcare or youth assistance	65 (229)	24.4	18.1–32.0
	Working in other sector	339 (1230)	23.8	21.0–27.0
	Not working	112 (450)	20.5	16.0–25.8
	Student	37 (133)	22.2	14.6–32.3
**Time to last vaccination**	Fully vaccinated- more than 3 months ago	501 (2565)	17.0	15.2–19.0
	Fully vaccinated- less than 3 months ago	251 (643)	32.7	28.1–37.6
	Partially vaccinated	60 (111)	47.8	36.5–59.3
**Previous positive COVID test**	No	713 (3002)	19.8	18.0–21.6
	Yes	99 (317)	25.8	19.9–32.8
**Communication of measures was good**	(totally) agree	422 (2216)	15.2	13.4–17.2
	neutral	214 (646)	27.9	23.7–32.6
	(totally) disagree	176 (457)	34.0	28.8–39.7
**Score effectiveness measures**^b^		18.7 (mean score)		18.2–19.4
**There are many people that I can count on completely**	(totally) agree	638 (2638)	20.4	18.5–22.5
	Neutral	114 (442)	19.4	15.2–24.3
	(totally) disagree	60 (239)	21.2	15.2–28.6

a Practical training is also referred to as vocational training, and theoretical training as college-bound.

b A high score indicates regarding COVID-19 infection prevention measures in September-December 2021 as ineffective.

Non-positive intention was higher in the age group 18–59 years (25.9%), compared to 60 and above (13.1%). Non-positive intention was voiced more often by females, compared to males (23.3% vs 16.2%). Booster vaccine intention differed between work situations. Non-positive intention was least often expressed by the retired group (11.4%), while those working in healthcare most frequently voiced non-positive intention (30.9%). Those with a negative experience with the communication of measures showed higher non-positive intention (34.0%) than those who had a neutral (27.9%) or a positive attitude (15.2%). Similarly, a negative attitude towards the effectiveness of infection prevention measures was more frequently observed in those with non-positive intention (mean score 18.7), compared to the group with positive intention (mean score 14.9).

### Logistic regression analysis

Assessing intention to take a booster vaccine by univariate logistic regression revealed significant differences between countries (Table 2). These differences were still present when correcting for other factors in the multivariable model. Statistically significant factors in the multivariable model included country, sex, absence of comorbidities, time to last vaccination, experience with communication of measures, and opinion on effectiveness of measures. Compared to German residents, Dutch (OR = 2.41; 95% CI = 1.90–3.04) and Belgian residents (OR = 1.40; 95%CI = 1.05–1.86) were more likely to be uncertain or unwilling to receive a booster vaccine. Female sex (OR = 1.55; 95%CI = 1.18–2.03), absence of comorbidities (OR = 1.33; 95% CI = 1.03–1.71), a neutral (OR = 1.64; 95% CI = 1.22–2.21) or negative (OR = 2.17; 95%CI = 1.56–3.02) experience with communication of COVID-19 measures, and regarding measures as ineffective (OR = 1.09; 95%CI = 1.06–1.11) were independently associated with non-positive intention. Those partially vaccinated (OR = 3.62; 95%CI = 1.98–6.63) and those fully vaccinated who received their last vaccine<3 months ago (OR = 1.61; 95%CI = 1.18–2.19) were more likely to have a non-positive intention.

**Table 2:** Weighted univariate and multivariable logistic regression models examining factors associated with non-positive intention.^a^

		**Crude OR**	**95% CI**	**p**	**Adjusted OR**	**95% CI**	**p**
**Country**	Germany	**1**		**<0.001**	**1**		**<0.001**
	Netherlands	**2.78**	**2.27**–**3.41**		**2.41**	**1.90**–**3.04**	
	Belgium	**1.45**	**1.12**–**1.87**		**1.40**	**1.05**–**1.86**	
**Age group**	≥60	**1**		**<0.001**	1		0.17
	18–59	**2.32**	**1.82**–**2.94**		1.30	0.89–1.90	
**Sex**	Male	**1**		**<0.001**	**1**		**0.002**
	Female	**1.57**	**1.25**–**1.99**		**1.55**	**1.18**–**2.03**	
**Level of education**^b^	Practical	1		0.24	1		0.56
	Theoretical	1.14	0.92–1.41		1.08	0.84–1.39	
**Work situation**	Retired	**1**		**<0.001**	**1**		**0.050**
	Working in healthcare	**3.48**	**2.34**–**5.17**		**1.86**	**1.07**–**3.24**	
	Working in education, childcare or youth assistance	**2.51**	**1.58**–**3.98**		1.10	0.61–1.99	
	Working in other sector	**2.44**	**1.78**–**3.33**		1.24	0.79–1.94	
	Not working	**2.00**	**1.34**–**2.99**		1.50	0.95–2.37	
	Student	**2.22**	**1.25**–**3.96**		0.73	0.34–1.54	
**Comorbidities**	Yes	**1**			**1**		**0.030**
	No	**1.62**	**1.30**–**2.01**	**<0.001**	**1.33**	**1.03**–**1.71**	
**Time to last vaccination**	Fully vaccinated- more than 3 months ago	**1**		**<0.001**	**1**		**<0.001**
	Fully vaccinated- less than 3 months ago	**2.37**	**1.84**–**3.06**		**1.61**	**1.18**–**2.19**	
	Partially vaccinated	**4.46**	**2.75**–**7.23**		**3.62**	**1.98**–**6.63**	
**Previous positive COVID test**	No	1		0.055			
	Yes	1.42	0.99–2.02				
**Communication of measures was good**	(totally) agree	**1**		**<0.001**	**1**		**<0.001**
	neutral	**2.17**	**1.66**–**2.84**		**1.64**	**1.22**–**2.21**	
	(totally) disagree	**2.89**	**2.17**–**3.84**		**2.17**	**1.56**–**3.02**	
**Score effectiveness (high score = don’t find measures effective)**^c^	per 1 unit change	**1.12**	**1.10**–**1.14**	**<0.001**	**1.09**	**1.06**–**1.11**	**<0.001**
**There are many people that I can count on completely**	(totally) agree	1		0.89			
	neutral	0.94	0.68–1.28				
	(totally) disagree	1.05	0.69–1.59				

a OR of ‘‘uncertainty or unwillingness” vs ‘‘willingness” responses of intention to take a booster vaccine.

b Practical training is also referred to as vocational training, and theoretical training as college-bound.

c 1 unit change indicates an increase of 1 in score. A high score indicates regarding COVID-19 infection prevention measures in September-December 2021 as ineffective.

## Discussion

This is the first study to assess COVID-19 booster intention in the EMR border region comparing the Netherlands, Belgium, and Germany. Intention for booster vaccination differs between the subregions of the EMR with the Netherlands showing the lowest intention, followed by Belgium, and Germany showing the highest intention for booster vaccination. Female sex, absence of comorbidities, time since the last COVID-19 vaccination less than 3 months ago or being partially vaccinated, a negative experience with communication of COVID-19 measures, and regarding measures as ineffective were independently associated with non-positive intention.

We assessed booster intention in a group of fully or partially vaccinated adults. Eventually, booster vaccination uptake rates in September 2022 reached 77% in Nordrhein-Westfalen, to which the German part of the EMR belongs, between 68% (Ostbelgien and Wallonia, to which Liège belongs) and 85% (Flanders, to which Belgian Limburg belongs) in the Belgian part of the EMR, and 63% in South Limburg, the Dutch part of the EMR [15], [16], [17]. Notably, similar differences between countries were observed for booster intention measured in our study and booster uptake, underscoring the validity of our study.

### What can we learn from each other?

Even though the EMR is a small region and people live at only a few kilometers distance from each other, differences exist between countries. This could be explained by differences in policies regarding COVID-19 and vaccination, as well as compliance with policies. In our study, citizens from the German side of the EMR were most positive about and adherent to the COVID-19 measures, whereas compliance was lowest in the Netherlands. Similarly, in other studies Germany showed a higher rating than other countries regarding the usefulness of measures and COVID-19 communication by the government was perceived as clear and understandable [32]. In Belgium, we observed larger differences in intention between different provinces. Nonetheless, this supports findings from another study on COVID-19 vaccine willingness among Belgians, where French-speaking participants were favorable to vaccination in general but less willing to be vaccinated against COVID-19 [33]. The COVID-19 pandemic proves the necessity of cross-border cooperation**.** It is recommended to keep up regular exchange and learn from each other, also outside of a pandemic situation. Cross-border care should be given more attention in national policy making. A large potential lies in exchanging protocols, organizing collaborative exercises, and supporting each other with resources [14].

### Determinants of non-positive booster intention

The finding that female sex and absence of comorbidities are associated with non-positive intention is consistent with other studies [8], [34], [10], [11], [12], [13]. The proportion of healthcare workers was relatively large in our study, and this group was more likely to have a non-positive intention of taking the booster vaccine. We had no data on employment status of the population in the EMR and thus could not include this factor in our weighting. However, COVID-19 vaccination and booster hesitancy in this group has been reported several times [35], [36].

We as well as other studies showed perceived effectiveness of measures and the experience of communication of measures by the government to be associated with booster vaccine intentions [9], [10], [37]. These factors are related to trust, which includes interpersonal trust, governmental trust, and trust in information [10], [38]. To measure interpersonal trust, we asked participants whether they have many people they can count on completely. In our study, no statistically significant effect was found, though interpersonal trust was associated with vaccine coverage in a study of 177 countries [38] In this study, high levels of government and interpersonal trust, as well as less government corruption, were associated with higher COVID-19 vaccine coverage. Public trust in the government can be strengthened by effective, consistent, and transparent communication [39]. Finally, lack of trust in vaccine information, related to an ‘infodemic’ forms another threat for vaccine acceptance. Susceptibility to misinformation has also been linked to lower COVID-19 risk perception [40], [41], indicating that policies focusing on modifiable risks such as battling misinformation and increasing governmental trust in general may be helpful in increasing COVID-19 vaccination intentions.

## Strengths and limitations

Differences in policies in the different countries at time of data collection likely affected the results of our study. However, a strength is that data were collected at the same time point in the three countries and booster intentions were asked exactly at the time when booster vaccination became or was about to become available. People might have participated in the study because of the incentive of getting to know their antibody status. However, bias may be present given that people who participate in research might also have a more positive attitude towards COVID-19 vaccination, which was illustrated by the high vaccination rate of the participants, compared to the general population at that time. A strength is that analyses were weighted and thus more representative of the general population of the EMR. Differences in booster vaccine intention between the three countries remained apparent after correcting for other factors. Nevertheless, the variables we included only partially explain the differences in booster vaccine intention between the countries. Other factors that we did not measure or were not able to measure could be responsible for the differences observed. Predictive factors identified in other studies on booster vaccine intention involved cultural differences, marital status, socioeconomic status, political orientation, religion, ethnicity, smoking status, and optimism [7], [9], [10], [11]. Also, people’s social network can influence decisions about health and vaccination [42].

A limitation of this study is that we did not ask participants’ reasons behind their booster vaccine intention, since this could provide a basis for interventions. Given that all participants in our study were vaccinated against COVID-19 at least once, reasons for non-positive intention might not have been related to skepticism towards COVID-19 vaccines in general. A report from April 2022 from the National Institute for Public Health and the Environment in the Netherlands reported on the most frequently cited reasons to not take a booster vaccine among 46,441 participants [43]. A commonly cited reason was that vaccination cannot continue forever and that the first series should give enough protection. It was also argued that vaccines are not the solution for the pandemic and booster vaccines are only effective for a short time. Some respondents indicated that they have enough trust in their own immune system or that booster vaccination is not necessary after experiencing a SARS-CoV-2 infection.

## Implications

It has been suggested that data and evidence should play a larger role in decision making, and that countries should comply with international health regulations during the pandemic. Comparable cross-border data collection allows for evaluation of different infection prevention policies on public health. An important step to prepare for future infectious disease outbreaks is committing to a treaty that keeps the issue elevated. This way, collaboration between countries could be facilitated and better coordinated [44]. Countries should be committed to work together to improve pandemic response in the future. Greater efforts are needed to ensure that the pandemic, including new variants, cases, and impact on the healthcare and other sectors remains manageable, partly through booster vaccination. Therefore, an important practical implication includes taking in consideration the situation in border areas. Besides, this study indicates which groups of the population should be focused on to reduce booster vaccine hesitancy.

## Conclusions

Booster vaccine intentions differ between countries in the EMR. Similar differences were observed in the eventual booster vaccine uptake, underscoring the validity of our study. Other factors associated with non-positive booster vaccine intention in this study were female sex, absence of comorbidities, time since last vaccination, and opinion on efficacy and communication of measures. The existence of differences between countries in this region highlights the need for cross-border collaboration to merge information and knowledge about vaccination strategies and ultimately limit the impact of COVID-19.

## Funding

The project was granted by the Interreg V-A EMR programme (NR. EMR166). The funder had no role in study design, data collection and analysis, decision to publish, or preparation of the manuscript.

## Data availability statement

The data of this study contain potentially identifying and sensitive participant information. Due to the General Data Protection Regulation, it is not allowed to distribute or share any personal data that can be traced back (direct or indirect) to an individual. In addition, publicly sharing the data would not be in accordance with participants’ consent obtained for this study. Therefore, data used and/or analyzed during the study are available from the head of the data-archiving of the Public Health Service South Limburg on reasonable request. Interested researchers should contact the head of the data-archiving of the Public Health Service South Limburg (Helen Sijstermans: helen.sijstermans@ggdzl.nl) when they would like to re-use data.

## Ethics approval and consent to participate

The study protocol, questionnaires, participant information form and written informed consent form were reviewed and approved by the Medical Ethical Committee of the MUMC+ (2020-2463-A-1). In Belgium, the study was approved by the Medical Ethical Committee of the University Hospital Ghent and the University of Ghent (BC-09754). In Germany, no ethical assessment of the project was required. The project was approved by the heads of Gesundheitsamt Heinsberg, Düren, and Aachen, and the in-house lawyers of Kreisverwaltung Heinsberg, Düren, and Städteregion Aachen. All participants provided fully informed consent.

### CRediT authorship contribution statement

**Céline J.A. van Bilsen:** Methodology, Formal analysis, Investigation, Writing - original draft. **Christina Stabourlos:** Investigation, Writing - review & editing. **Chrissy P.B. Moonen:** Investigation, Writing - review & editing. **Stephanie Brinkhues:** Funding acquisition, Conceptualization, Methodology, Formal analysis, Investigation, Writing - review & editing. **Stefaan Demarest:** Funding acquisition, Conceptualization, Methodology, Writing – review & editing. **Daniëlle A.T. Hanssen:** Funding acquisition, Conceptualization, Writing – review & editing. **Inge H.M. van Loo:** Funding acquisition, Conceptualization, Writing – review & editing. **Paul H.M. Savelkoul:** Funding acquisition, Conceptualization, Writing – review & editing. **Dirk Philippsen:** Funding acquisition, Conceptualization, Writing – review & editing. **Brigitte A.M. van der Zanden:** Funding acquisition, Conceptualization, Writing – review & editing. **Nicole H.T.M. Dukers-Muijrers:** Funding acquisition, Conceptualization, Methodology, Formal analysis, Writing – review & editing. **Christian J.P.A. Hoebe:** Funding acquisition, Conceptualization, Methodology, Formal analysis, Writing - review & editing.

## Declaration of Competing Interest

The authors declare that they have no known competing financial interests or personal relationships that could have appeared to influence the work reported in this paper.

## Data Availability

Data will be made available on request.
